# Functional networks in prolonged disorders of consciousness

**DOI:** 10.3389/fnins.2023.1113695

**Published:** 2023-02-17

**Authors:** Hui Li, Xiaonian Zhang, Xinting Sun, Linghui Dong, Haitao Lu, Shouwei Yue, Hao Zhang

**Affiliations:** ^1^Cheeloo College of Medicine, Shandong University, Jinan, Shandong, China; ^2^Department of Neurorehabilitation, China Rehabilitation Research Center, Beijing, China; ^3^University of Health and Rehabilitation Sciences, Qingdao, Shandong, China

**Keywords:** prolonged DoC, network, functional connectivity, fMRI, neuroimaging

## Abstract

Prolonged disorders of consciousness (DoC) are characterized by extended disruptions of brain activities that sustain wakefulness and awareness and are caused by various etiologies. During the past decades, neuroimaging has been a practical method of investigation in basic and clinical research to identify how brain properties interact in different levels of consciousness. Resting-state functional connectivity within and between canonical cortical networks correlates with consciousness by a calculation of the associated temporal blood oxygen level-dependent (BOLD) signal process during functional MRI (fMRI) and reveals the brain function of patients with prolonged DoC. There are certain brain networks including the default mode, dorsal attention, executive control, salience, auditory, visual, and sensorimotor networks that have been reported to be altered in low-level states of consciousness under either pathological or physiological states. Analysis of brain network connections based on functional imaging contributes to more accurate judgments of consciousness level and prognosis at the brain level. In this review, neurobehavioral evaluation of prolonged DoC and the functional connectivity within brain networks based on resting-state fMRI were reviewed to provide reference values for clinical diagnosis and prognostic evaluation.

## Introduction

The incidence of disorders of consciousness (DoC) has increased sharply due to the development of first-aid and intensive care techniques over the years. DoC is characterized as states of unconsciousness induced by severe brain injuries involving trauma (O'Donnell et al., 2019; Giacino et al., 2020), hemorrhage (Crone et al., 2014), or hypoxic–ischemic encephalopathy such as cardio-pulmonary resuscitation (Weng et al., 2017; Peran et al., 2020) or poisoning (De Paepe et al., 2012). The temporal division of DoC includes the acute phase from a few days or weeks after brain injury when patients get treated in the emergency room or intensive care unit, with the addition of subacute and chronic phases when patients spent time in a rehabilitation center, care facilities, or home (Edlow et al., 2021). Subsequently, prolonged DoC was used to describe subacute and chronic phases of patients ≥28 days following the primal brain injury (Giacino et al., 2018), including vegetative state (VS)/unresponsive wakefulness syndrome (UWS) and minimally conscious state (MCS; Schnakers, 2020). With an in-depth understanding and continuous evolution, the recognition of a locked-in syndrome (LIS) state (cognitively intact but complete or near-complete paralysis) and non-behavioral MCS (MCS star or MCS^*^, patients in the VS/UWS state who may preserve partial brain activities that resemble those in MCS) have provided a more precise distinction between patients in a comatose state and conscious-wakefulness (Hocker and Wijdicks, 2015; Thibaut et al., 2021).

It is generally accepted that the cumulative effect of differentiation of central thalamic neurons and active inhibition of neocortical and striatal neurons leads to extensive regression of synaptic activity and low cerebral metabolic rates, ultimately generating a range of unresponsive symptoms in patients with DoC (Thibaut et al., 2019; Edlow et al., 2021; Zheng et al., 2022). Meanwhile, the recovery of consciousness is regarded as closely relevant to the restoration connections within corticothalamic neuronal activity (Wagner et al., 2020; Edlow et al., 2021). Based on these theories, resting-state functional magnetic resonance imaging (fMRI) is recommended as part of the clinical multimodal evaluation and provides valuable information for brain networks to detect those possibly subtle transformations in brain activities (Snider and Edlow, 2020; Norton et al., 2023).

In this review, we discuss the clinical behavioral evaluations of prolonged DoC and target studies that investigated the correlation between prolonged DoC and separate brain networks. To explore their diagnostic and evaluation value in patients with prolonged DoC, we searched PubMed for articles published in English between 1 January 2012 and 31 October 2022 using the following search terms: “consciousness disorders[Mesh],” and “fMRI,” “network,” or “assessment.” Seven major brain networks involved “default mode network,” “salience network,” “executive control network,” “dorsal attention network,” “auditory network,” “visual network,” and “sensorimotor network” (Raichle, 2011, 2015). We screened clinical trials, case reports, and review articles that included patients with prolonged DoC and were relevant to the topic. Additional references were collected and reviewed from the included articles' bibliographies.

## Neurobehavioral evaluation of prolonged DoC

Accurate diagnosis of prolonged DoC is not only necessary for the medical teams to make prognosis estimation but also provides meaningful information and helps family members participate in valid clinical care support and clinical decision-making. However, a misdiagnosis rate of 30–40% was reported from consensus-based expert diagnoses (Schnakers et al., 2009), including misdiagnoses of those that have emerged from the vegetative state into a VS/UWS or LIS into a VS or an MCS (van Erp et al., 2015; Vanhaudenhuyse et al., 2018). Here are a few possible reasons. First, the performance of patients with prolonged DoC fluctuated incessantly, especially when some inconsistent responsiveness could only be elicited *via* certain stimulation or in specific situations. Second, measurement outcomes could be largely influenced by the patient's own disease or complications (e.g., cranial nerve palsies, quadriplegia, severe spasticity, and dystonia). In addition, the assessor's experience (lack of extended observation of patients or under training) may also have led to considerable reporting bias and error in the results (Childs et al., 1993). It follows that limited clinical examination may lead to an underestimation of consciousness levels in patients in a VS/UWS or an MCS, and the diagnostic accuracy of bedside qualitative examination needs to be enhanced.

The American Congress of Rehabilitation Medicine reviewed a number of neurobehavioral scales for DoC that have been applied to diagnose and predict functional outcomes (Seel et al., 2010). Of these, the most accepted and recommended was the Coma Recovery Scale-Revised (CRS-R), which includes six subscales—audition, vision, motion, mouth movement, communication, and arousal level—and is widely used to diagnose and classify different levels of consciousness owing to its reliable validity and reliability (Tamashiro et al., 2014; Binder et al., 2018; Han et al., 2018; Iazeva et al., 2018; Zhang et al., 2019). In addition, the Full Outline of Unresponsiveness Score (FOUR) showed substantial evidence of good interrater reliability and could reduce the misdiagnosis of locked-in syndrome and MCS for patients in the intensive care unit (Kondziella et al., 2020). The Sensory Modality Assessment Technique (SMART; da Conceicao Teixeira et al., 2021), Western Neuro Sensory Stimulation Profile (WNSSP; Cusick et al., 2014), Sensory Stimulation Assessment Measure (SSAM; Park and Davis, 2016), Wessex Head Injury Matrix (WHIM; Shiel et al., 2000), and Disorders of Consciousness Scale (DOCS; Pape et al., 2014) are recommended for assessing DoC with moderate reservations. Rather, the Coma/Near-Coma Scale (CNC; Weaver et al., 2021) may be suitable for patients with major reservations. Although standardized behavioral assessment scales might outperform clinical expert diagnosticians' bedside evaluation for signs of consciousness (Schnakers et al., 2009), even a single assessment of CRS-R might result in a misdiagnosis rate of 36% in patients with prolonged DoC (Wannez et al., 2017). The accuracy rating of these diagnosis scales is still limited due to the battery of confusion factors in patients' and assessors' experiences listed earlier.

## Neuroimaging and electrophysiological assessment

To date, diverse auxiliary inspection tools have been used in the diagnosis and assessment of prolonged DoC. Positron emission tomography (PET) was first used to identify preserved but covert cortical processing evidence in patient in VS (Menon et al., 1998). The application of PET provides evidence for cortical activation in patients with prolonged DoC and helps to identify different unconsciousness states (Laureys and Schiff, 2012). By contrast, the electroencephalogram technology (EEG) method is widely applicable and could also provide objective information for the evaluation efficacy of patients with prolonged DoC, especially appropriate for bedside inspection (Chennu et al., 2017). EEG-derived neuronal signals including both speech-tracking responses and temporal dynamics of whole-brain neuronal networks were reported to be related to the behavioral diagnosis of consciousness and wakefulness prediction (Gui et al., 2020; Zhang et al., 2022). Continuous EEG and quantitative EEG could also provide effective value for diagnosis and initial consciousness recovery (Hwang et al., 2022; Lutkenhoff et al., 2022). In addition, functional near-infrared spectroscopy (fNIRS) is another non-invasive method to quantitatively detect brain function based on cerebral oxygen information in real time (Kempny et al., 2016).

Compared with EEG and fNIRS, fMRI has higher spatial resolution and better integration with structural lesions and is more available than PET (Ansado et al., 2021). Assessment during the resting state is particularly opportune for patients with prolonged DoC since patient interaction and application of possible experimental setups are mostly difficult and infeasible. Recent studies have measured the brain's spontaneous neural activities by resting-state fMRI (rs-fMRI), which used blood-oxygen-level-dependent (BOLD) contrast to reflect fluctuations and uncover the important process underlying consciousness (Palanca et al., 2015; Zhang et al., 2021). The BOLD signal was thought to provide an indirect measure of brain function that is closely related to ongoing neuronal events in the brain (Phillips et al., 2016) and could be used to forecast human behavior (Ward et al., 2020). The superiority of its sensitivity and technical simplicity have made other non-invasive imaging techniques of fMRI outshone (Jann et al., 2015). Of note, it has been suggested that spontaneous BOLD fluctuation is not random but specifically correlated with the spatially distinct systems and brain networks in the resting human brain (Keller et al., 2011). It is, thus, possible that functional connectivity, measured by the BOLD signal, is disturbed in brain networks in prolonged DoC.

The intrinsic activities of the brain are linked to multiple temporal and spatial-related functional networks through integrating structural or functional connections of different cortical regions. It has been reported that the brain networks of prolonged DoC changed from that when in a comatose state until they recovered consciousness (Cavanna et al., 2018; Threlkeld et al., 2018; Crone et al., 2020) and potentially predicted recovery (Wu et al., 2015; Zou et al., 2017; Zhang et al., 2018). Moreover, the detectable rate of intrinsic cortical activity in MCS seems higher than that in a coma or VS/UWS with resting-state fMRI (Kondziella et al., 2020). This suggests that the whole-brain dysfunction after brain injury may underlie the abnormal network connectivity of prolonged DoC, which is strongly correlated with the level of consciousness. Moreover, by calculating functional temporal correlations within spatially separated neurophysiologic activities from fMRI, functional connectivity could be used to identify covert signatures of consciousness in patients with prolonged DoC and reflect the inherent brain activities (Bodien et al., 2019; Snider and Edlow, 2020).

## Functional networks in prolonged DoC

**Figure 1:**
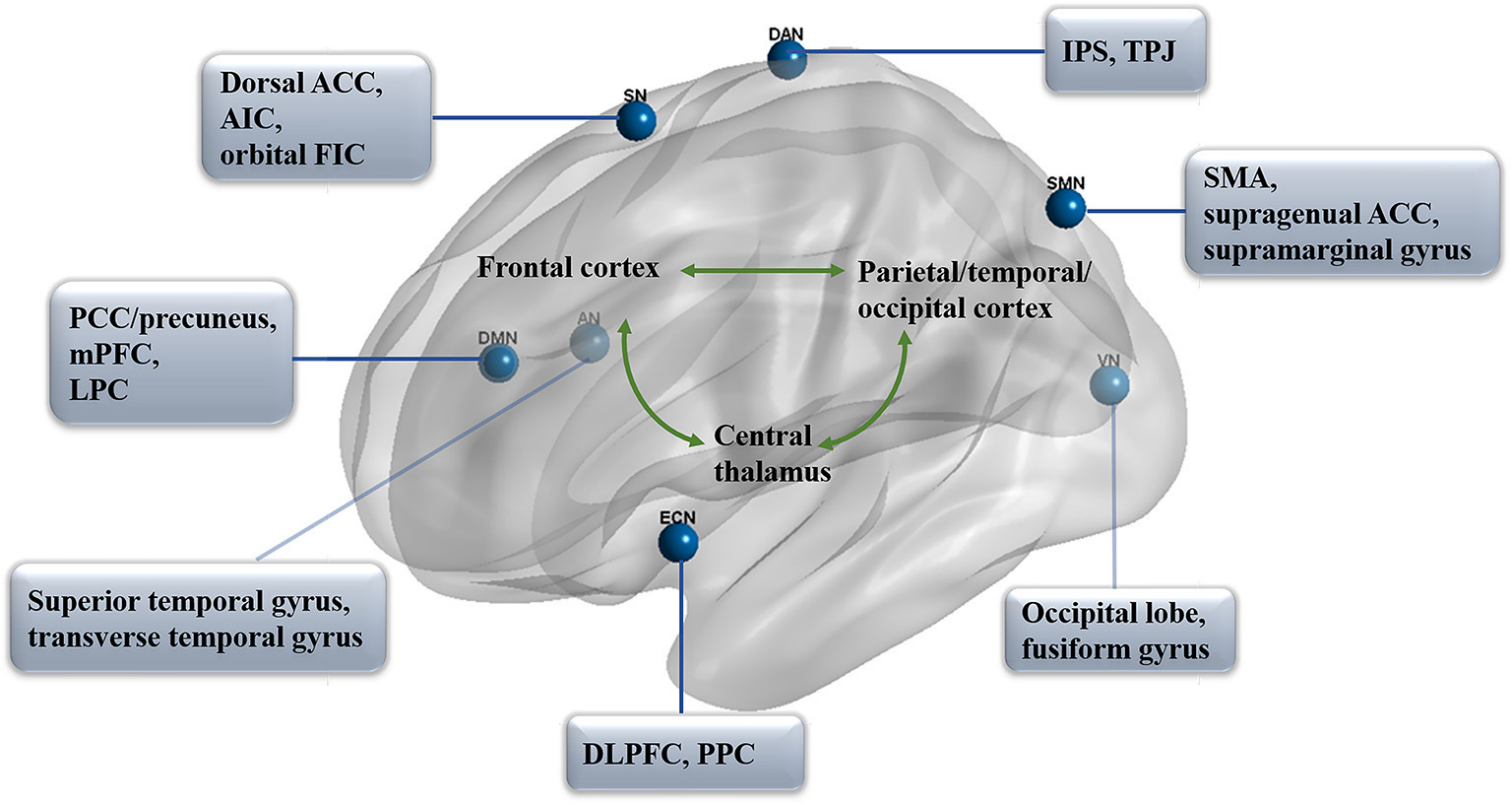
Functional networks in prolonged DoC. The distribution of seven primary networks that control the resting state of functional connectivity in prolonged DoC. DMN, default mode network; PCC, posterior cingulate cortex; mPFC, medial prefrontal cortex; LPC, lateral parietal cortex; DAN, dorsal attention network; IPS, intraparietal sulcus; TPJ, temporoparietal junction; ECN, executive control network; DLPFC, dorsolateral prefrontal cortex; PPC, posterior parietal cortex; SN, salience network; ACC, anterior cingulate cortex; AIC, anterior insula cortex; FIC, frontoinsular cortices; AN, auditory network; VN, visual network; SMN, sensorimotor network; SMA, supplementary motor area.

As we know, two primary positively correlated components are involved in consciousness, wakefulness, and awareness (Naro et al., 2017). In particular, awareness can be subdivided into two parts: environment (external) and self (internal) awareness (Demertzi et al., 2011). It has been identified that the default mode network (DMN) exhibits internal activities, also referred to as the “task-negative network” (Andrews-Hanna, 2012; Andrews-Hanna et al., 2014), whereas the lateral frontoparietal areas related to the network of dorsal attention (DAN; Mallas et al., 2021) and executive control (ECN; Martin-Signes et al., 2019) mediate task-driven stimuli (Xin et al., 2021; i.e., task-positive network). These two sets of regions are reported to be negatively correlated with healthy adults, anesthetic patients, or patients with prolonged DoC (He et al., 2014; Palanca et al., 2015), both under resting-state or attention-demanding processes (Lyu et al., 2021). In another case, according to the regulating function, the brain networks could be classified into higher order networks [the DMN, ECN, DAN, and salience network (SN)] and sensory-related (perceptual processing) lower order networks including the sensory input auditory network (AN; Braga et al., 2017), visual network (VN; Wang Y. et al., 2020), and sensorimotor network (SMN; Liang et al., 2015; Figure 1). Notably, altered functional brain networks have been observed in different types of unconsciousness states, such as in deep sleep (Samann et al., 2011; Boly et al., 2012; Houldin et al., 2021; Rue-Queralt et al., 2021; Tarun et al., 2021), anesthesia (Qiu et al., 2017; Golkowski et al., 2019; Malekmohammadi et al., 2019; Wang S. et al., 2020), pathological hypnosis (Cojan et al., 2015; McGeown et al., 2015; Jiang et al., 2017), and psychedelics (Tagliazucchi et al., 2016; Preller et al., 2019; Luppi et al., 2021a). As for patients with prolonged DoC, the functional connectivity in key regions of each network was reported to be correlated with CRS-R scores from different distributions and functions (Demertzi et al., 2015).

## Default mode network (DMN)

The default mode network contains a set of brain regions that are more active during the resting state than when they focus on features of the external environment, such as attention-demanding tasks (Buckner and Krienen, 2013; Raichle, 2015). Compared with the regions of the cortex that is more directly constrained by extrinsically driven neural activity, the DMN took on roles that are both more complex and less directly influenced. This network is active in internally oriented mentation such as “mind-wandering,” “daydreaming,” or “self-referential processing” (Konishi et al., 2015; Yeshurun et al., 2021). To date, the DMN has been the most studied network in prolonged DoC, and its functional connectivity is not only critical for the detection of consciousness levels but also involved in the process of awareness emergence in these patients (Fernandez-Espejo et al., 2012; Norton et al., 2012; Crone et al., 2015; Qin et al., 2015a). The within-network correlations were recognized as positive DMN connectivity, and anti-correlations between networks were recognized as negative DMN connectivity (Di Perri et al., 2016). Functional connectivity within the DMN was found to be decreased in patients with DoC, ranging from those in an MCS and UWS to those in a coma state (Fernandez-Espejo et al., 2012; Norton et al., 2012; Crone et al., 2015; Hannawi et al., 2015; Coulborn et al., 2021), and remained intact in patients with locked-in syndrome (Vanhaudenhuyse et al., 2010).

Generally, there are three major fields in the DMN: the medial prefrontal cortex (mPFC), the posterior cingulate cortex (PCC), and the adjacent precuneus plus the lateral parietal cortex (LPC; Leech and Sharp, 2014; Raichle, 2015), which constitute the primary intrinsic functional connectivity in patients with DoC (Wu et al., 2015). The neuropathological basis of the DoC includes the interruption of connections within the DMN, which involves key regions, such as PCC and mPFC (Silva et al., 2015). The mPFC is a large, complex, and heterogeneous area with the highest baseline metabolic activity at rest (Gusnard et al., 2001) and could be broadly classified into distinct subregions along with the dorsal to the ventral axis: the medial precentral area, anterior cingulate cortex (ACC), prelimbic cortex (PL), and infralimbic cortex (IL; Xu et al., 2019). Among these regions, some researchers have suggested that the dorsal medial prefrontal cortex (dmPFC) contains the dorsal region of the ACC and the PL, while the ventral PL, IL, and dorsal peduncular cortex belong to the ventral medial prefrontal cortex (vmPFC; Jasinska et al., 2015). Resting-state activity in the mPFC was regarded as correlated with private self-consciousness (Huang et al., 2016). Of which, functional activation of the dmPFC subsystem was considered, specifically associated with rumination (Zhou et al., 2020) and perceptual memory (Schwiedrzik et al., 2018). By contrast, as the sensory-visceromotor component of the DMN, the vmPFC plays a role in theory-of-mind ability, processing self-relevant information, and greater extinction memory in humans' ability to modulate fear (Hebscher et al., 2016; Hiser and Koenigs, 2018; Nejati et al., 2021). Fast network oscillations are consistently larger in the dmPFC than in the vmPFC region in anesthetized animals (Gretenkord et al., 2017), which reflects possible different inputs to mPFC subregions in prolonged DoC.

Intrinsic functional connectivity strength in the PCC/precuneus was found to be significantly correlated with consciousness level, recovery outcome, and differential diagnosis (Bonfiglio et al., 2014; Palhano-Fontes et al., 2015; Flamand et al., 2018). The PCC serves as a main connector hub within functional neural distinct networks in the DMN and plays an important role in integrating the neural representations of self-location, body ownership, and internally directed thoughts (Leech et al., 2012; Guterstam et al., 2015). As sleep depth increased, contributions of the PCC and mPFC to the DMN seem to be decreased (Samann et al., 2011). In addition, it was suggested that the PCC is the only DMN node that interacts with most of the other DMN nodes and is strongly co-activated with the mPFC (i.e., dmPFC and vmPFC; Fransson and Marrelec, 2008; Supekar et al., 2010). This region is characterized by the BOLD signal time series during rest conditions and is distinguished from task-positive network regions (Yu et al., 2011). Notably, patients in VS showed significantly reduced self-inhibition and increased oscillations in the PCC compared with those of patients in MCS and healthy people (Crone et al., 2015). Furthermore, the DMN may be related to the prognosis prediction of patients with prolonged DoC. It was evident that PCC and left LPC connectivity differentiate patients with UWS who recovered consciousness after 3 months from those who did not (Qin et al., 2015a), and patients in coma exhibit significantly enhanced functional connectivity in the PCC/precuneus when they regained consciousness (Norton et al., 2012; Guo et al., 2019). These findings indicate that as a relatively independent network module, the functions of the brain regions within DMN are closely connected, and the PCC/precuneus and mPFC in DMN are found to be important brain network hubs in prolonged DoC (Silva et al., 2015; Wang et al., 2019).

## Salience network (SN)

The salience network contributes to the identification of stimulus processing that guides behavior (Heine et al., 2012; Miyata, 2019), attention control (Peters et al., 2016), or interoceptive awareness/conscious perception (Chong et al., 2017; Ueno et al., 2020). This network could also be activated by interoceptive stimuli as part of a representation of all feelings from the body, such as pain (Veréb et al., 2020). The SN comprises the dorsal ACC, the bilateral anterior insula cortex (AIC), and the orbital frontoinsular cortices (FIC) and has connections to subcortical regions, including the amygdala, the substantia nigra/ventral tegmental area, the thalamus, and the limbic structures (Veréb et al., 2020). It was reported that AIC, especially the anterior and ventral (inferior) areas, are involved in the representation of all subjective feelings from both body and emotional awareness, such as self-recognition and time perception (Craig, 2009), and play a fundamental role in human awareness. The SN is non-uniformly impaired in unconsciousness states, such as in anesthetic (Bonhomme et al., 2016; Golkowski et al., 2019), psychedelic (Lebedev et al., 2015), or epileptic states (Lee et al., 2018). Similar to the DMN, the reduced functional connectivity in this key network is also correlated with the degree of impaired consciousness in prolonged DoC. Functional connectivity of the SN is reduced in patients in MCS but hardly identified in patients in VS/UWS (Demertzi et al., 2015). Moreover, the functional connectivity between the AIC and ACC may also play a fundamental role in awareness (Luo et al., 2014) and emotional feelings (Krach et al., 2015). Compared with MCS, patients in UWS showed significantly reduced functional connectivity between supragenual ACC and left AIC within the SN (Qin et al., 2015a).

In addition, the SN may also serve as a “switch” between the “task-positive” network and the “task-negative” network (Goulden et al., 2014). First, the SN and the “task-positive” network DAN are anti-correlated with DMN, including, the SN and DAN having an inhibitory influence on DMN regions, whereas the DMN in turn excites SN and DAN regions (Zhou et al., 2018). In addition, functional connectivity between the SN and another “task-positive” network ECN was observed to be positively elicited under hypnosis (Jiang et al., 2017), and it was reported that an anesthetic-induced unresponsive state generates small increases in bidirectional connectivity within the SN and ECN (Ihalainen et al., 2021). Moreover, the structural and functional integrity of the SN seems to be necessary for efficient regulation of the activity of the DMN. The structural damage in the SN may specifically predict abnormalities in DMN function (Bonnelle et al., 2012), and stimulus inherent salience could attenuate the deactivation BOLD responses of the PCC in the DMN, which could be offset by a sufficient level of glutamate in the dorsal ACC (von Düring et al., 2019). Therefore, it is reasonable to presume that the SN is a potential neural correlate of consciousness.

## Executive-control network (ECN)

As stated, awareness is related to a large-scale frontoparietal network that comprises two distinct subsystems in processing the self and external-related components of awareness (Haugg et al., 2018). In the composition of awareness, except for the impaired DMN that is involved in internal awareness, the ECN acts more like a lateral and dorsal frontoparietal network involved in the awareness of the environment and related to externally guided awareness (Luppi et al., 2021b). It is centered on the dorsolateral prefrontal cortex (DLPFC) and the posterior parietal cortex (PPC), and also includes the frontal eye fields (FEF) and part of the dorsomedial prefrontal cortex (dmPFC) that coordinate executive function (Chen et al., 2013; Friedman and Robbins, 2022; Menon and D'Esposito, 2022). This network regulates behavioral measures of executive control (e.g., attention, working memory, and cognitive control), including the voluntary control of novel and complex situations (Martin-Signes et al., 2019). Moreover, the anterior ECN was reported to be involved in interference control, which modulates perceptual sensitivity and conscious perception (Colás et al., 2017). Previous studies suggested that there are neural correlates between executive control and conscious perception in frontal–parietal regions by functional connection analysis (Martin-Signes et al., 2019; Martín-Signes et al., 2021). Compared with the wake state, the within-network functional connectivity of the DMN, SN, and ECN was observed to be significantly reduced under unresponsive states (drug sedation or deep sleep; Guldenmund et al., 2017). In addition, fewer patients in MCS and VS/UWS showed components of neuronal origins for bilateral ECN compared with healthy controls (Demertzi et al., 2014). It could be speculated that the ECN constitutes a crucial neural substrate of the global workspace that enables consciousness control. Moreover, it has been suggested that the reduced functional connectivity between the DLPFC and precuneus enables the former a popular therapeutic target for non-invasive brain stimulation in prolonged DoC as to restore the disrupted balance between the ECN and DMN (Qin et al., 2015b; O'Neal et al., 2021).

## Dorsal attention network (DAN)

The dorsal attention network (DAN) is a vital part of the “task-positive” network and typically modulates brain activity to exert control over thoughts, feelings, and actions during task performance (Humphreys and Sui, 2016; Lu et al., 2019). The DAN could be subdivided into endogenous and exogenous control components. The endogenous attention control components link the dorsal frontoparietal regions and cover the intraparietal sulcus (IPS), while exogenous components are associated with the ventral frontal and temporoparietal regions, including the temporoparietal junction (TPJ; Bourgeois et al., 2013; Ahrens et al., 2019). It was reported that functional connectivity within the DAN was reduced under anesthetic-induced light sedation (Wang et al., 2021). Moreover, the DAN is also negatively correlated with the DMN and constitutes negative DMN connectivity (Fox et al., 2005; Favaretto et al., 2022). In comparison, the DMN mediates the recurrence of thoughts experienced during past events, whereas the DAN may contribute to the visuospatial attention distribution of episodic memory features (Stawarczyk et al., 2018). As the anti-correlation between the spontaneous activity of the DMN and DAN increased, patients' behavioral performance became more consistent, and these negative correlations seem to be decreased proportionally under anesthesia (Boveroux et al., 2010). It was reported that the switching between these two networks is crucial for conscious cognition and might be a more credible marker for tracking alterations of consciousness even than the positive DMN connectivity in patients with prolonged DoC (Di Perri et al., 2016). In any event, the disruption in both positive DMN connectivity and negative DMN connectivity seemed always to be increased with the improvement of consciousness (i.e., from UWS, MCS, and emergence from MCS to healthy controls; Boly et al., 2009; Di Perri et al., 2016).

## Auditory, visual, and sensorimotor networks

As is well-known, the direct clinical diagnosis of prolonged DoC is mainly based on the behavior responses reflected from auditory, visual, and sensorimotor cortices (Kondziella et al., 2020). The visual system consists of the primary, lateral, and occipital visual networks including the occipital lobe and the fusiform gyrus (Heine et al., 2012; Wang Y. et al., 2020). Interestingly, there were studies suggesting that the activity and connectivity in lower order networks appear to be less affected under unresponsive states, while higher order brain networks are significantly weakened (Boveroux et al., 2010; Kirsch et al., 2017; Wang et al., 2021). For instance, the functional integrity of higher order networks was severely disrupted by light sedation when lower level networks were found to be globally preserved (Liang et al., 2015). Patients in MCS might also preserve large-scale cortical networks associated with language and visual processing (Giacino et al., 2006). However, other studies suggested that the functional activities of low-order networks in prolonged DoC are reduced, especially in patients in VS/UWS, and all these networks have a certain capacity to discriminate against patients with prolonged DoC (Demertzi et al., 2015; Medina et al., 2022). Specifically, decreased connectivity between visual and SMN (Amico et al., 2017) and ECN (Mikell et al., 2015) was observed in unresponsive patients, respectively. The exact reason is not clear, but we suspect that the inconsistent results may be partly due to different etiologies or inducements, as well as different analysis methods of brain networks.

In contrast, more studies have explored AN elicited by voice stimulation under task-state fMRI, which may be related to the prognosis of prolonged DoC (Di et al., 2007; Wang et al., 2015). Nevertheless, the functional connectivity of AN at the resting state could also be used to distinguish patients in an MCS from those in a VS/UWS, and the reduced connectivity between the auditory and visual cortices may be more sensitive to distinguish patients independently (Boveroux et al., 2010; Demertzi et al., 2015). This might be partly due to the disrupted anatomical connections in patients with DoC and the direct comparison between patients in MCS and VS/UWS among these networks. The regions of the AN encompassed the bilateral auditory cortices including the superior/transverse temporal gyrus and are associated with TPJ (Laureys et al., 2000; Demertzi et al., 2014). Auditory–visual functional connectivity is considered relevant to multisensory integration, which is indispensable in predicting forthcoming sensory events and differentiating patients with prolonged DoC (Boly et al., 2008). In particular, according to the analysis of network neuronal properties (neuronal vs. non-neuronal), the DMN and AN were thought to discriminate patients from healthy subjects with high accuracy (Demertzi et al., 2014).

Apart from the primary somatosensory and ventrobasal thalamic nucleus that transmits somatosensory cortical activity, and the primary motor cortex and the ventral lateral thalamic nucleus that carry motor control information (Kang et al., 2016), there is a higher order sensorimotor circuit of the brain's global functional network that supports consciousness in the sensorimotor processing. This circuit is constituted by the supplementary motor area (SMA), the supragenual ACC, the bilateral supramarginal gyrus, and the left middle temporal gyrus (Qin et al., 2021). Prior studies have shown abnormal activities or connectivity in higher order sensory and motor regions in patients in a UWS, healthy people who are asleep, or patients under anesthesia (Mitra et al., 2015; Qin et al., 2021), while the stimulus-evoked activity of primary sensory regions is largely preserved.

## Discussion

As fMRI has been increasingly applied in the clinical utility and investigation of neurological diseases, its clinical values in prolonged DoC are increasingly significant (Albrechtsen et al., 2022). Previous studies have mainly applied fMRI to the baseline consciousness assessment and brain function exploration in prolonged DoC (Crone et al., 2014; Weng et al., 2017; Zhang et al., 2018), providing insights into the neural mechanisms of brain networks that have not been fully understood so far. In addition to brain injury, functional connectivity and network integrity are also disturbed to varying degrees in aging (Malagurski et al., 2020; Patil et al., 2021) and neurodegenerative disorders including mild cognitive impairment, Alzheimer's disease, Parkinson's disease, and amyotrophic lateral sclerosis (Zhu et al., 2021; Miao et al., 2022; Thome et al., 2022; Zhao et al., 2022). Of which, the DMN is highly vulnerable. The underlining mechanisms remain unclear so far, but some studies suggested that the DMN is especially vulnerable to amyloid deposition (Hampton et al., 2020; Guzman-Velez et al., 2022) and inconsistently activated across time (Malagurski et al., 2020).

Here, we focused on the major brain networks that have been identified as being associated with prolonged DoC in the last few years. Based on this research, it is determined that prolonged DoC is associated with severely impaired resting state network connectivity, especially in higher order (Demertzi et al., 2015; Kirsch et al., 2017). Notably, numerous studies have indicated that the impaired functional connectivity within the brain networks is present in a consciousness-level-dependent manner (Norton et al., 2012; Crone et al., 2015; Panda et al., 2022; Wang et al., 2022), even in linear correlation (Di Perri et al., 2016), and most networks seem to have a high discriminative capacity to separate patients in an MCS and VS/UWS (Demertzi et al., 2015). Of the seven networks we listed, the DMN is the most concerned brain network in prolonged DoC. The functional connectivity strength between the mPFC and PCC/precuneus has potentially significant value for the prediction of consciousness awakening (Norton et al., 2012; Guo et al., 2019). In addition, the negative DMN connectivity including the anti-correlation between the spontaneous activity of the DMN and DAN or ECN was also found to be impaired in prolonged DoC (Boly et al., 2009; Qin et al., 2015b; Di Perri et al., 2016), as well as the SN may play an important role in switching between “task-positive” and “task-negative” networks (Goulden et al., 2014; Zhou et al., 2018).

Apart from consciousness assessment and supportive diagnosis, recently, fMRI was applied as an evaluation tool to estimate the therapeutic efficacy of wake-promoting treatment such as transcranial direct current stimulation (Aloi et al., 2022), transcutaneous auricular vagus nerve stimulation (Yu et al., 2017), zolpidem (Rodriguez-Rojas et al., 2013), sensory stimulation (Pape et al., 2015), amantadine, and transcranial magnetic stimulation (Bender Pape et al., 2020). In particular, abnormal functional connectivity as assessed by resting-state fMRI is pivotal in personalized target identification in neuromodulation therapy (Ren et al., 2022). This means that fMRI may be an effective technique to assist in the treatment of prolonged DoC. However, this still needs a lot of research to confirm. In addition, given that DLPFC is one of the most commonly used targets for neuromodulation therapy in patients with prolonged DoC (O'Neal et al., 2021), ECN may also serve as an important network for efficacy evaluation as well as DMN.

Furthermore, although increasing research has been devoted to exploring the brain networks of prolonged DoC, few studies have delved into the different etiologies. A previous study analyzed the fMRI data of 29 patients with cardiac arrest and 14 patients with traumatic brain injury, the results indicated that posteromedial cortex disturbance was particularly found in patients with cardiac arrest, whereas cingulum architectural was found in traumatic patients (Peran et al., 2020). However, the relationship between functional networks and different pathological states of the brain remains poorly understood. Future studies are required to elucidate differences in functional connectivity between prolonged DoC of different etiologies, as well as between patients with prolonged DoC, medicated sedation, or in deep sleep states to facilitate more accurate diagnosis and the development of personalized treatment. Moreover, it is worth noting that there is a major challenge facing the application of fMRI in prolonged DoC, that is, most of these patients are inapposite for MRI scanning. Whether it is the intracranial metal, large areas of brain tissue deformation, or the unconscious head movement during the process, would all limit the clinical practice and data analysis of fMRI in prolonged DoC (Desai et al., 2015; Kirsch et al., 2017). Future compatible technologies and advanced algorithms are expected to overcome and improve this problem.

## Conclusion

In recent years, the study of neurofunctional imaging in the field of DoC has evolved from small sample-based studies on areas-of-interest networks to multicenter across whole-brain network studies, which significantly advanced our understanding of the brain function in patients with prolonged DoC at the network level, allowing them to be dynamically modeled gradually. Meanwhile, due to the numerous analytical methods of fMRI, one or two reports are sometimes insufficient to be fully replicated. Further investigations might aim at larger samples of patients and provide more objective and cautious evidence.

## Author contributions

HL designed and wrote the original draft. XZ, XS, and LD aided in literature retrieval and screening. HL and HZ were involved in writing-review and editing. SY and HZ supervised and administrated the study. All authors contributed to the article and approved the submitted version.
