# Corrigendum: Functional networks in prolonged disorders of consciousness

**DOI:** 10.3389/fnins.2023.1208095

**Published:** 2023-05-03

**Authors:** Hui Li, Xiaonian Zhang, Xinting Sun, Linghui Dong, Haitao Lu, Shouwei Yue, Hao Zhang

**Affiliations:** ^1^Cheeloo College of Medicine, Shandong University, Jinan, Shandong, China; ^2^Department of Neurorehabilitation, China Rehabilitation Research Center, Beijing, China; ^3^University of Health and Rehabilitation Sciences, Qingdao, Shandong, China

**Keywords:** prolonged DoC, network, functional connectivity, fMRI, neuroimaging

In the published article, there was an error in affiliation for Hao Zhang. Instead of “Hao Zhang^1,2,3^,” it should be “Hao Zhang^2,1,3^.” There was an error in affiliation 1. Instead of “Rehabilitation Center, Qilu Hospital, Cheeloo College of Medicine, Shandong University, Jinan, Shandong, China”, it should be “Cheeloo College of Medicine, Shandong University, Jinan, Shandong, China.”

The authors apologize for this error and state that this does not change the scientific conclusions of the article in any way. The original article has been updated.

